# Optical coherence tomography-assisted interventional treatment of a woven coronary artery: a case report

**DOI:** 10.1186/s12872-023-03233-8

**Published:** 2023-04-21

**Authors:** Wei Wei, Qi Zhang, Liming Gao

**Affiliations:** Department of Cardiovascular Medicine, East Hospital, Tongji University School of Medicine, 150 Jimo Road, Shanghai, 200120 China

**Keywords:** Optical coherence tomography, Woven coronary artery, Recanalization

## Abstract

**Background:**

A woven coronary artery is a rare feature on coronary angiography. Distinguishing woven-like change and recanalization of an occluded artery is difficult.

**Case presentation:**

A 59-year-old man was admitted to the hospital for chest tightness. Coronary angiography showed a woven coronary artery at the middle segment of the left anterior descending branch (LAD). The middle segment of the right coronary artery (RCA) exhibited 99% occlusion. A 3.5 × 26-mm drug-eluting stent was implanted in the middle segment of the RCA. One week later, we examined the LAD using optical coherence tomography (OCT). Considering the patient’s medical history and the results of OCT, a 2.7 × 38-mm drug-eluting stent was inserted into the LAD. Re-examination by OCT indicated that the stent was well attached to the wall, with no dissection at the edge of the stent. Antiplatelet therapy and statin-based plaque stabilization were continued. No obvious abnormality was found during follow-up one year later.

**Conclusions:**

OCT can help distinguish woven-like change and recanalization of an occluded coronary artery. The evaluation and formulation of treatment strategies for a woven coronary artery are important.

## Background

A woven coronary artery is a rare feature observed on coronary angiography. It has been considered a benign congenital structural abnormality [1]; however, recent studies have reported that its presence may lead to unstable angina, acute myocardial infarction, and even sudden death [2–4]. Distinguishing woven-like change and recanalization of an occluded artery using coronary angiography is difficult.

## Case presentation

A 59-year-old man was admitted to the hospital on March 15, 2021, for chest tightness and shortness of breath of 7 days’ duration. An untreated acute myocardial infarction had occurred 12 years previously. The patient had a history of hypertension and chronic kidney disease, both stable under management. Physical examination revealed the following: an alert mental state; heart rate, 80 beats/min; regular rhythm; and no audible murmurs. Auscultatory lung sounds were unremarkable. No edema was present in the lower limbs. Examination for myocardial infarction after admission revealed the following: cardiac troponin T, 0.691 ng/mL; creatine kinase-MB isoenzyme, 5.25 ng/mL; pro-brain natriuretic peptide, 1822 ng/L; serum creatinine, 161.2 µmol/L; and serum uric acid, 638.2 µmol/L. Electrocardiography revealed sinus rhythm, ST segment abnormalities (0.5–2.0-mm depression in leads I, II, V3, V4, V5, and V6), and T wave alterations. The preliminary diagnoses were coronary heart disease, acute non-ST-elevation myocardial infarction, old myocardial infarction, Killip class I cardiac function, high-risk stage 3 hypertension, and chronic kidney disease.

**Fig. 1:**
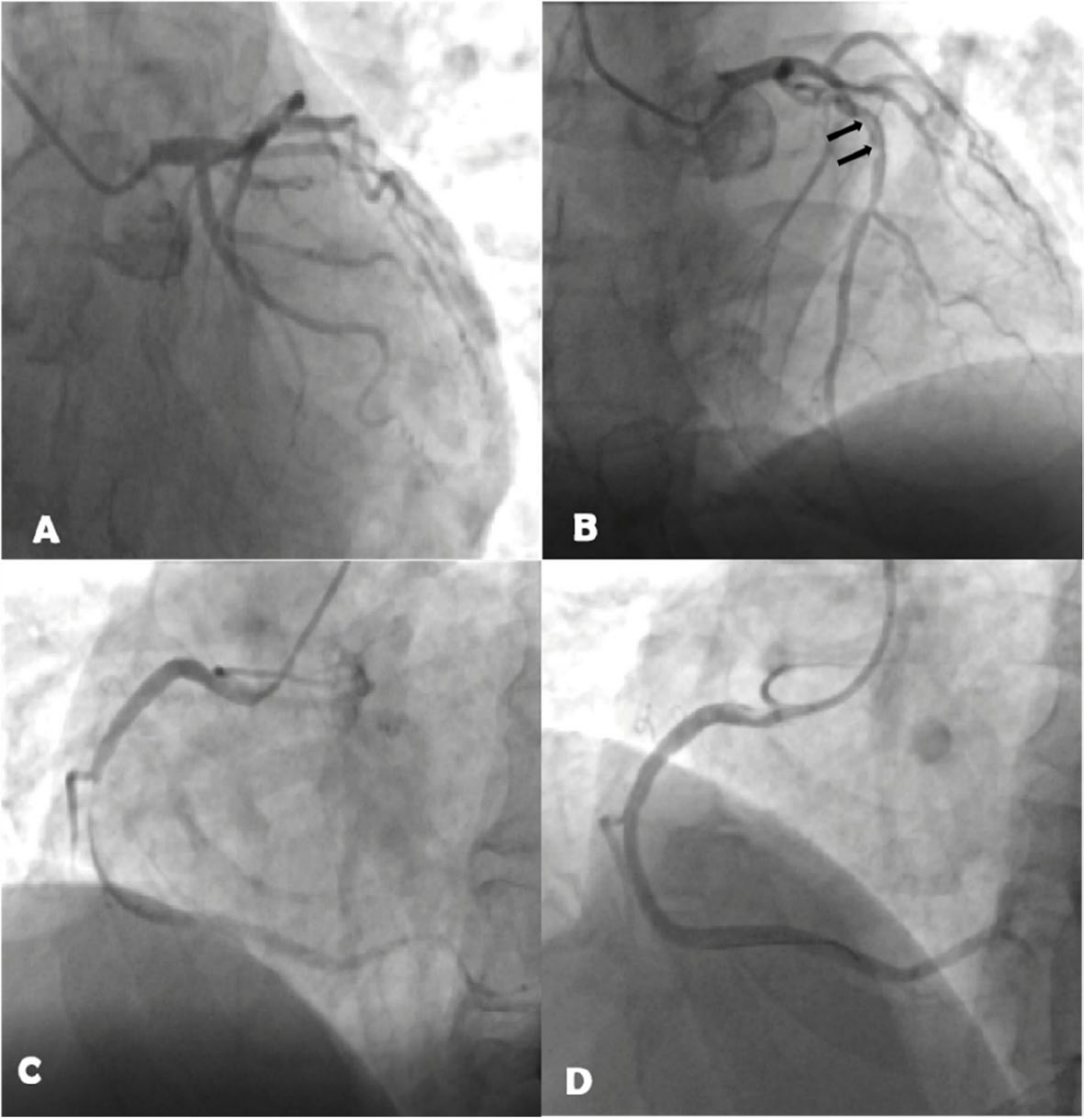
After the first intervention. **A**, no stenosis of the left circumflex artery; **B** (arrow), woven coronary artery anomaly in the middle segment of the left anterior descending artery; **C**, occlusive lesion in the middle segment of the right coronary artery (RCA); **D**, after stent placement in the middle segment of the RCA

Elective coronary angiography (Fig. 1) showed a right-dominant pattern and no obvious stenosis in the left coronary artery. A woven coronary artery anomaly, 10–15 mm in length, was present in the middle segment of the left anterior descending (LAD) branch. The distal segment of the LAD had 30–40% localized stenosis and Thrombolysis In Myocardial Infarction (TIMI) flow grade 3 (Fig. 1A, B). The origin of the first diagonal branch had 90% localized stenosis and TIMI flow grade 3 (Fig. 1B). The circumflex artery had no obvious stenosis and TIMI flow grade 3. The middle segment of the right coronary artery (RCA) exhibited 99% occlusion distal to the acute marginal branch, with visible thrombosis and distal TIMI flow grade 2 (Fig. 1C). Considering the patient’s symptoms and the results of coronary angiography, the RCA was considered to be the artery related to the infarction. A 3.5 × 26 mm drug-eluting stent with 99% occlusion (Resolute Integrity) was implanted in the middle segment of the RCA (Fig. 1D).

**Fig. 2:**
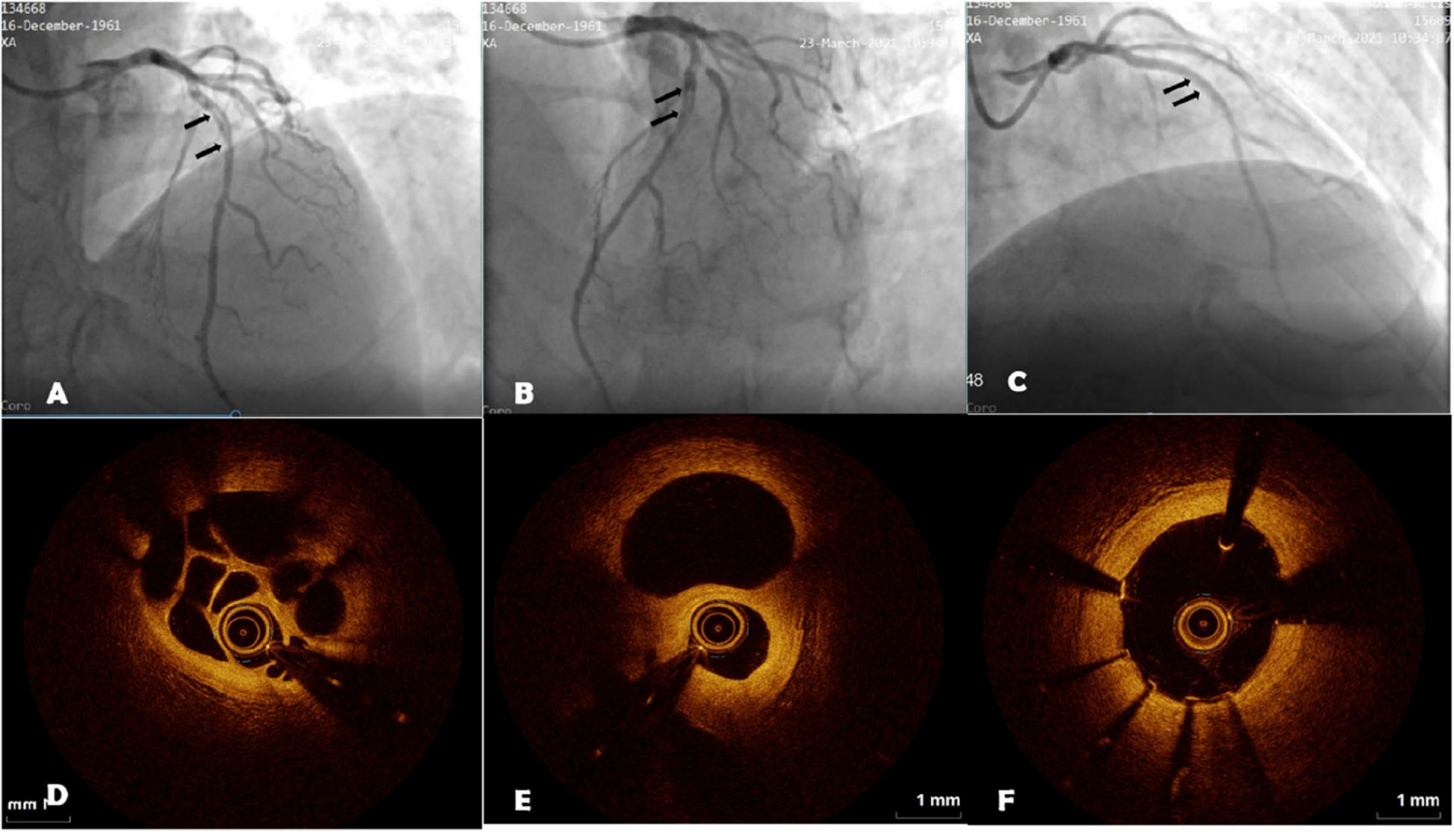
Second intervention and the optical coherence tomography (OCT) process. **A**, **B**, (arrow) woven coronary artery; **C**, left anterior descending stent implantation; **D**, **E**, preoperative OCT images of the woven coronary artery; **F**, post-percutaneous coronary intervention OCT image

Coronary angiography was repeated one week later. The LAD anomaly was unchanged (Fig. 2A, B), and the RCA stent was unobstructed. Optical coherence tomography (OCT) was planned for the LAD anomaly, with percutaneous coronary intervention (PCI) if necessary. The intervention was then performed as follows (Fig. 2): A 6 F EBU 3.5 guiding catheter (Medtronic Inc., Dublin, Ireland) was placed in the LAD opening, and a Fielder-XT hydrophilic-coated guide wire (Asahi Intecc USA, Inc., Irvine, CA, USA) was smoothly passed through the anomaly, entering the distal LAD. OCT showed that the guide wire was positioned within the lumen, and the intima, media, and adventitia were visible. There were honeycomb-like changes in the anomaly, and part of the false cavity was covered by the intima. Considering the patient’s medical history and OCT results, the cause was believed to be recanalization of the organized thrombus after the previous myocardial infarction (Fig. 2D, E). PCI was performed using a pre-expanded 2.0 × 15 mm Firefighter balloon catheter inserted into the proximal segment of the anterior descending branch. The catheter was expanded at 10 atm for 5 s, and a 2.7 × 38 mm drug-eluting stent (Helios) was inserted into the proximal anterior descending branch, expanded at 10 atm for 5 s, and released. The pre-expanded 2.0 × 15 mm balloon catheter was then inserted into the D1 opening and expanded at 10 atm for 5 s; then noncompliant 2.75 × 10 mm and 3.0 × 10 mm balloons were inserted into the stent and expanded at 12–14-16 atm for 5 s. There was no residual stenosis in the stent (Fig. 2C). Re-examination indicated that the stent was well attached to the wall, there was no dissection at the edge of the stent (Fig. 2F), and TIMI flow grade was 3.

The patient did not experience discomfort after the operation. Subsequent monitoring of myocardial enzyme levels showed dynamic changes consistent with myocardial infarction. There were no changes in renal function, and the patient was discharged on March 25, 2021, after which combined antiplatelet therapy and statin-based plaque stabilization were continued. No obvious abnormality was found during the follow-up one year later.

## Discussion and conclusions

It is rare to encounter a woven-like change in one coronary artery and an acute coronary syndrome caused by a different culprit coronary artery. There are no pre-existing reports of single vessel acute thrombotic lesion, where a special woven-like change was found in a different vessel. Using OCT in this case, we found that the woven-like change was caused by the recanalization of an occluded artery. A 2017 autopsy report of a sudden-death case suggested a woven coronary artery as the cause of death. That case first revealed the histopathological characteristics of a woven coronary artery, suggesting that each narrow vessel has a three-layered vascular structure, and the vessels are not interconnected, thus raising major doubts about whether it is indeed a benign variation [3]. Furthermore, recanalization of an organized thrombus in the coronary artery and spontaneous spiral dissection of the coronary artery can also resemble a woven coronary artery on coronary angiography [5–7], as it did in our case. The treatment strategies and prognosis for a woven coronary artery greatly differ with different etiologies. The development of intracoronary imaging, particularly intravascular OCT, has facilitated the identification of the specific etiology of this imaging feature and has guided its treatment.

Compared to intravascular ultrasound, OCT can distinguish the structure of the vessel wall more clearly and has significant advantages in distinguishing between true and false lumina, intramural hematomas, and intraluminal thrombi [8]. Many researchers believe that a woven coronary artery is a benign congenital anomaly that rarely causes thrombotic events [4–7], and many woven coronary artery anomalies are diagnosed using coronary angiography alone. OCT can improve the diagnostic accuracy for this anomaly [9, 10]. Studies have shown that a woven coronary artery is a feature on coronary angiography, and that OCT displays an intact vascular lumen instead of several independent small vascular lumina [6], whereas other reports mention honeycomb or Swiss cheese-like features on OCT [11–13]. This OCT feature tends to indicate recanalization of an organized thrombus. In the present case, the old myocardial infarction and the lesions on OCT clarified that the woven coronary artery was caused by recanalization of an organized thrombus.

Currently, the reasons for the recanalization of an organized thrombus are unclear; however, it is certain that the distal blood flow is insufficient in most cases [11], and the small available luminal area may lead to decreased distal blood flow, leading to acute myocardial infarction. Case reports have indicated that such an organized thrombus with a woven appearance may lead to chronic coronary occlusion if untreated, and may cause part of the thrombus to detach and embolize to the distal blood vessels during the unstable recanalization process [12]. Our case reports for the first time the woven-like changes that occur during acute myocardial infarction. The stability, or lack thereof, of the disease and the vessel with myocardial infarction affect the prognosis of the patient. For an unstable organized thrombus with a woven appearance, intracoronary interventional therapy is necessary.

The treatment strategies for a woven coronary artery remain debatable. In our case, interventional therapy was successful because the OCT allowed us to confirm the stable state of the patient and select an appropriate route. However, situations are often more complicated. Many researchers believe that a woven coronary artery does not affect blood flow and only requires regular follow-up. However, many previously reported cases [5, 6] of woven coronary arteries had intrinsically atypical convergences of multiple small vascular lumina, and the possibility of recanalization of an organized thrombus could not be ruled out. Based on intravascular imaging, interventional therapy may be considered for recanalization of an organized thrombus confirmed by OCT. However, there is no clear evidence that interventional therapy can significantly reduce the incidence of major adverse cardiac events in such diseases. On the one hand, the management of stable organizing thrombotic lesions depends on blood flow rather than the lesion itself. On the other hand, there are often many difficulties in interventional treatment, as the guide wire must pass through narrow pores in a long, hard lesion, which is technically challenging demanding and associated with a risk of dissection. In addition, the branching blood vessels at the lesion site are easily lost, and the probability of the branch guide wire entering a false lumen is high. Therefore, the recanalization of an organized thrombus in a woven coronary artery is quite challenging. Intravascular imaging such as OCT is crucial for positioning the guide wire and distinguishing between true and false lumina. In the present case, the location of the guide wire within the true vessel lumen and the position of the branch guide wire were confirmed with the aid of OCT for accurate stent positioning, thereby reducing the risk of plaque displacement. After implantation and full expansion of the stent, OCT re-examination was performed to confirm the expansion and immobilization of the stent, and to evaluate edge dissection, which is also particularly important in a woven coronary artery [13]. Of course, OCT is not completely risk-free in such lesions. In a previous report, after the guidewire was successfully extended to the distal end of the lesion in a woven coronary artery and the OCT probe tip was passed through the lesion, ventricular fibrillation was immediately induced [14]. This may be related to the temporary blockage of blood flow when the available luminal area is too small. Therefore, formulating interventional treatment strategies for a woven coronary artery is still extremely challenging, and the risks must be fully assessed in advance, in combination with appropriate intravascular imaging. Unlike previous cases, the use of OCT in this case not only confirms the need for a guide wire within the lumen prior to stent placement, but also distinguishes the woven phenomenon. In this case, woven change is defined as the recanalization of the organized thrombus. From the perspective of "monism", it suggests that this patient is a recurrent ASCVD case, and that both vessels have been affected by atherosclerosis, which requires interventional treatment to improve long-term prognosis.

In general, there is currently no uniform opinion on whether a woven coronary artery is a benign variation, and there is no consensus on its treatment strategies. However, it is clear that OCT plays an important role in identifying the nature of the disease, formulating treatment strategies, positioning the guidewire, positioning stent implantation, and evaluating stent expansion and edge dissection. OCT guidance is recommended for the evaluation and formulation of treatment strategies for a woven coronary artery. Of course, the addition of fractional flow reserve for evaluating the distal blood flow of relatively stable lesions may allow treatment plans to be optimized, thus yielding greater benefits.

## Data Availability

Not applicable.

## Abbreviations

LAD: The left anterior descending branch
OCT: Optical coherence tomography
PCI: Percutaneous coronary intervention
RCA: Right coronary artery
TIMI: Thrombolysis In Myocardial Infarction
